# Nanotip Contacts for Electric Transport and Field Emission Characterization of Ultrathin MoS_2_ Flakes

**DOI:** 10.3390/nano10010106

**Published:** 2020-01-04

**Authors:** Laura Iemmo, Francesca Urban, Filippo Giubileo, Maurizio Passacantando, Antonio Di Bartolomeo

**Affiliations:** 1 Department of Physics ‘E.R.Caianello’, University of Salerno, Via Giovanni Paolo II, 132-84084 Fisciano (SA), Italy; liemmo@unisa.it (L.I.); furban@unisa.it (F.U.); 2 CNR-SPIN Institute, Via Giovanni Paolo II, 132-84084 Fisciano (SA), Italy; filippo.giubileo@spin.cnr.it; 3 Department of Physical and Chemical Science, University of L’Aquila, via Vetoio, Coppito, 67100 L’Aquila, Italy; maurizio.passacantando@aquila.infn.it

**Keywords:** molybdenum disulfide, field effect transistor, field emission, contacts, electric transport

## Abstract

We report a facile approach based on piezoelectric-driven nanotips inside a scanning electron microscope to contact and electrically characterize ultrathin MoS_2_ (molybdenum disulfide) flakes on a SiO_2_/Si (silicon dioxide/silicon) substrate. We apply such a method to analyze the electric transport and field emission properties of chemical vapor deposition-synthesized monolayer MoS_2_, used as the channel of back-gate field effect transistors. We study the effects of the gate-voltage range and sweeping time on the channel current and on its hysteretic behavior. We observe that the conduction of the MoS_2_ channel is affected by trap states. Moreover, we report a gate-controlled field emission current from the edge part of the MoS_2_ flake, evidencing a field enhancement factor of approximately 200 and a turn-on field of approximately 40 V/μm at a cathode–anode separation distance of 900 nm.

## 1. Introduction

Molybdenum disulfide (MoS_2_) is one of the most studied members of the transition metal dichalcogenide family [1,2] as an alternative to graphene [3,4] for new-generation electronic devices and sensors based on atomically thin 2D materials. It has been extensively used to develop field effect transistors (FETs) [5,6], photodetectors [7], photovoltaic cells [8], light emitters [9], integrated circuits [10], field emission devices [11,12], and chemical [13] or biological [14] sensors.

Mono- and few-layer MoS_2_ flakes can be produced either by mechanical exfoliation (from bulk material) [15], or can be synthesized by chemical vapor deposition (CVD) [16]. At present, for larger scale production, high-quality MoS_2_ flakes are preferably produced by CVD.

The MoS_2_ bulk form consists of a layered structure held by van der Waals interactions [17]. Three atomic layers with the molybdenum plane in between two sulfur planes constitute each monolayer. Bulk MoS_2_ presents an indirect bandgap (1.2 eV), which becomes direct and larger, up to 1.9 eV, for monolayers [18,19,20]. The high direct bandgap makes this material suitable for FET applications with an on/off ratio exceeding $10^{8}$ [21] and for selective light absorption in optoelectronic devices with high photoresponsivity [7]. Unfortunately, the carrier mobility for these devices is limited to a few tens of ${\mathrm{cm}}^{2}V^{-1}s^{-1}$ [22] due to short- and long-range scattering caused by the presence of structural defects, such as S (sulfur) vacancies, or Coulomb traps and surface corrugations related to the substrate [23,24,25].

Due to its sharp edges and high aspect ratio, few-layers MoS_2_ is also considered suitable for field emission (FE). Several technological applications require the controlled propagation of electrons in a vacuum, such as flat-panel displays, microwave amplifiers, electron microscopy, and X-ray sources. Field emission is a quantum mechanical tunneling process by which the electrons are extracted from a surface (metallic or semiconducting) by the application of an external electric field so the electrons can flow in a vacuum from a cathode to an anode. For a flat cathode, FE is enabled by a strong electric field (several kV/μm), while if the cathode surface has sharp edges or protrusions, electrons may be extracted by a considerably lower applied electric field, since the physical geometry provides a field enhancement near the emitting surface. To date, several nanostructures have been investigated as possible field emitters, like metallic nanowires and nanoparticles [26,27,28], semiconducting nanowires and nanoparticles [29,30,31,32,33], nanodiamonds [34], carbon nanostructures [35], carbon nanotubes (CNTs) [36,37,38,39,40,41]**,** and graphene [33,42,43,44]. Instead, few studies have investigated FE from MoS_2_ structures, such as sheets and nanosheets [45,46], nanotubes and nanoflowers [47,48], nanostructures [49], thin films [50]**,** and bilayers [12].

The deposition of metal electrodes on 2D materials by standard electron-beam lithography (EBL) and lift-off processes is an expensive step, in economic and time terms, during the device’s fabrication. In this work, we propose a facile method to easily realize metal contacts on MoS_2_ flakes that can be used for a first assessment of their suitability for FETs and FE devices. Such a method is based on the use of piezoelectric-driven W (tungsten) tips inside a scanning electron microscope (SEM) chamber, connected to external source-meter units, which are gently approached onto the flake under the combined control of SEM imaging and electrical current monitoring. Moreover, the method can provide a reliable electrical characterization without exposing the flake to process-related damage.

With such an approach, we analyze the electrical transport properties of CVD-synthesized *n*-type monolayer MoS_2_ FETs in a back-gate configuration and their modifications due to gate voltage sweeping range, sweeping time, and electron-beam irradiation. We investigate the effects of the trap centers on the gate hysteresis and we observe that the hysteresis width increases linearly with the gate voltage range and exponentially with the sweeping time. Furthermore, we demonstrate that after exposure to electron-beam irradiation, the *n*-doping of the channel increases. Considering the *n*-type doping, we can study the local FE properties from the edge part of the flake. We demonstrate that the gate voltage can modulate the FE current. At the cathode–anode distance of 900 nm, we find a maximum value of the field enhancement factor of 200 and a turn-on field of approximately 40 V/μm at the gate voltage of 40 V. This study demonstrates that it is possible to extensively and reliably characterize 2D material-based devices even without complex nanofabrication by EBL, metal sputtering/evaporation, and lift-off techniques. The proposed method enables a quick selection of flakes before engaging in the laborious production of field effect or field emission devices or the characterization of devices unaffected by process-related damages.

We note that a similar lithography-free method to perform electrical measurements of 2D materials, using a manually actuated microprobe station with carbon fiber microprobes for the direct electrical contact, has previously been proposed [51]**.** However, the method here demonstrated utilizes thinner probes (approximately 100 nm), fine-controlled by piezo-driven arms, which can be reliably placed with a separation less than 1 μm, thus enabling measurements even on very small flakes. Furthermore, the SEM imaging instead of the optical one allows higher-resolution monitoring of the contact regions.

## 2. Materials and Methods

**Figure 1:**
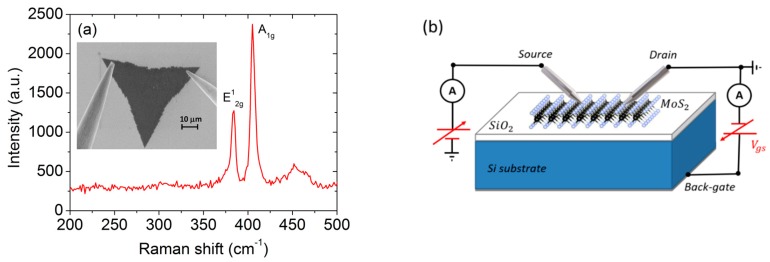
(**a**) Raman spectrum of the monolayer MoS_2_ flake. The inset displays a SEM image of the MoS_2_ flake and the two W-tips used as electrical contacts. (**b**) Schematic of the device and the measurement setup.

MoS_2_ flakes were synthesized by CVD at a temperature of 750 K, using S powder and a saturated ammonium heptamolybdate solution as precursors, on a SiO_2_/Si substrate. To evaluate the number of MoS_2_ layers, we performed micro-Raman spectroscopy measurements (using a 532 nm laser source). In Figure 1a, we report the Raman spectrum, evidencing the standard (in-plane) $E_{2g}^{1}$ and (out-of-plane) $A_{1g}$ vibration modes. The frequency separation of the two peaks of approximately $20{\;\mathrm{cm}}^{-1}$ indicates a monolayer flake [52,53]. The inset in Figure 1a shows a scanning electron microscopy (SEM) image of the flake and of the two sharp piezoelectric-driven tungsten tips (curvature radius approximately 100 nm) used as electrical contacts.

Electrical measurements were performed inside a SEM chamber (LEO 1530, Zeiss, Oberkochen, Germany) at room temperature and high vacuum ($10^{-6}\;\mathrm{Torr}$), using metallic tips mounted on piezoelectric-driven nanoprobes. Probes were electrically connected to a semiconductor parameter analyzer (Keithley 4200-SCS, semiconductor characterization system, Tektronix Inc., Beaverton, OR, USA), with a current sensitivity of $10^{-13}A$. The piezoelectric control of the probes allows the fine tuning of the movements with a spatial resolution of about 5 nm and enables a gentle and non-destructive approach on the flakes. The approach is performed with the following procedure. Using SEM imaging, the tips can be positioned above the flake with an incertitude of 50–100 nm. After that, one of the two tips is harshly pushed against the flake (by selecting an edge or a location of no interest) until a movement or a scratch is observed on it. After that, we gradually move the second tip in 5 nm steps and simultaneously monitor the current between the two tips. We systematically observe a sudden rise of the current from the noise floor of $10^{-13}A$ without any apparent damage of the approach point on the flake. Finally, we detach the first tip and repeat the gentle approach of it to a desired location.

The schematic layout of the device and the experimental setup is reported in Figure 1b. The silicon substrate was used as the back gate and metallic tips as the drain and the source electrodes for the FET characterization. For the field emission measurements, we exploited the same setup, using a tip as the cathode and retracting the other tip (anode) from the MoS_2_ flake at a controlled cathode–anode separation distance.

## 3. Results and Discussion

### 3.1. Transistor Characterization

**Figure 2:**
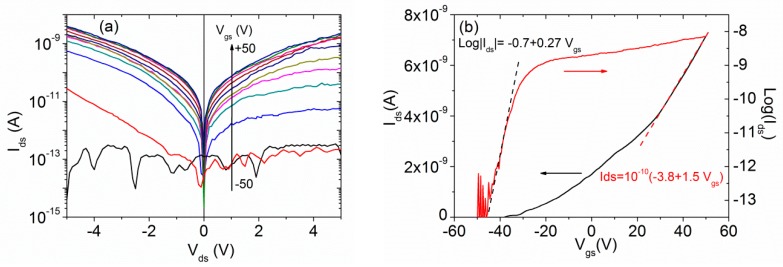
(**a**) $I_{ds}\;\;V_{ds}$ output characteristics of the MoS_2_ back-gated FET at different gate voltages $V_{gs}$, in steps of 10 V, with source-drain current on logarithmic scale. (**b**) $I_{ds}-V_{gs}$ transfer characteristics at $V_{ds}=-5\;V$ with source-drain current on logarithmic and linear scales, and linear fitting curves (dashed lines).

The electrical characterization by the tip-contact method of a selected MoS_2_ flake, used as the channel of a back-gated FET, is reported in Figure 2. The source-drain $I_{ds}-V_{ds}$ output characteristics as a function of the gate voltage $V_{gs}$, shown in Figure 2a, reveal a rectifying behavior, which can be attributed to the formation of asymmetric Schottky barriers between the channel and the contacts, as often occurs when contacting MoS_2_ [54,55]. Figure 2b shows the $I_{ds}-V_{gs}$ transfer characteristics at the drain bias $V_{ds}=-5\;V$, on a linear (black curve) and logarithmic scale (red curve). The FET reveals n-type behavior with a threshold voltage of $V_{T}\;\approx -10.5\;V$ ($V_{gs}$ corresponding to a $I_{ds}=\;1\;\mathrm{nA}$), indicating a n-doped channel, as reported in several studies due to the chemisorption of oxygen on MoS_2_ surface defects and sulfur vacancies [56,57,58].

The $I_{ds}$ current on the logarithmic scale of Figure 2b shows an on/off ratio greater than $10^{5}$ and a subthreshold swing of $SS=\frac{dV_{gs}}{dLog\left(I_{ds}\right)}\;\sim \;4\;\frac{V}{\mathrm{decade}}$, i.e., the gate-voltage variation due to the one-decade increase of the FET current. From the slope of the transfer characteristics on the linear scale of Figure 2b, we evaluated the mobility $\mu =\frac{L}{W}\frac{1}{C_{SiO_{2}}}\frac{1}{V_{ds}}\frac{dI_{ds}}{dV_{gs}}\;\sim \;1{\;\mathrm{cm}}^{2}V^{-1}s^{-1}$, where L and W are the channel length and width, respectively, and $C_{SiO_{2}}=11{\;\mathrm{nF}\;\mathrm{cm}}^{-2}$ is the capacitance per unit area of the SiO_2_ with a thickness of 300 nm. We assumed the distance between the tips (13 μm) and their diameter (200 nm) as the length and width of the transistor channel, respectively. The obtained mobility is within the range ($0.05-70{\;\mathrm{cm}}^{2\;}V^{-1}s^{-1}$) commonly reported in FETs with a MoS_2_ channel on SiO_2_ [22,59,60,61]. Its relatively low value can be caused partially by the high contact resistance but is mainly an indication of a high density of scattering centers, such as intrinsic defects in the crystal structure of MoS_2_, extrinsic traps at the MoS_2_/SiO_2_ interface or into the SiO_2_ dielectric layer, and charged impurities such as adsorbates on the MoS_2_ surface [6].

**Figure 3:**
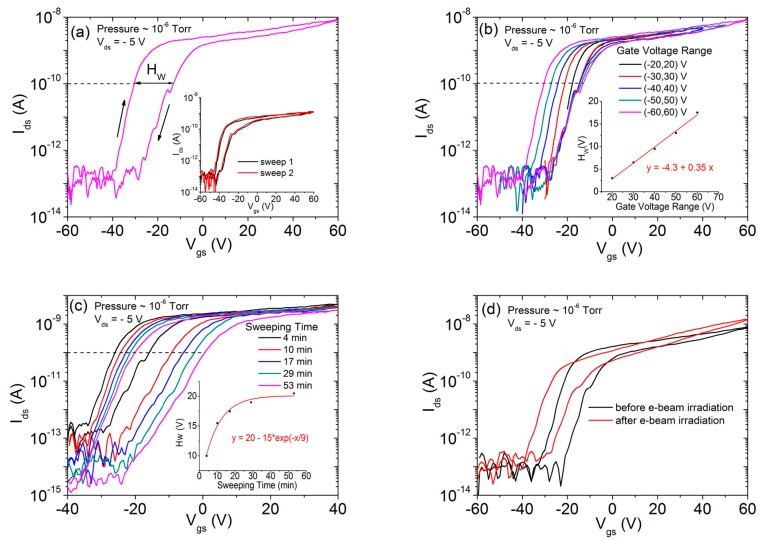
(**a**) Forward and backward transfer characteristics. The inset shows two transfer curves obtained before (sweep 1) and after (sweep 2) the two tips were detached and re-connected to check the reproducibility of the measurements. (**b**) Transfer characteristics (complete sweep loop) plotted as a function of the gate voltage range and (**c**) the sweeping time. The insets show the linear and the exponential dependence of the hysteresis width as a function of the gate voltage range and the sweeping time, respectively. (**d**) Transfer characteristics before and after electron-beam irradiation. All transfer characteristics were measured at $V_{\mathrm{ds}}=-5\;V\;$and at$\;10^{-6}\;\mathrm{Torr}\;$ pressure.

In Figure 3a, we report the transfer characteristics measured for a gate-voltage sweep between  60 V and 60 V (forth and back), which induces Joule heating on the device that has been in high vacuum (at $10^{-6}\;\mathrm{Torr}$) for a long time. The combined effect of Joule heating and low pressure reduces/removes adsorbates and makes it possible to analyze the effect of intrinsic defects and traps only. The complete sweeping shows a right shift of the transfer curve, creating a hysteresis that can be explained in terms of negative charge trapping [25,56,62]. Hysteretic behavior can be analyzed by the hysteresis width $H_{W}$ (i.e., the difference of the gate voltage values corresponding to a channel current $I_{ds}=\;0.1\;\mathrm{nA}$). Figure 3b,c shows two features of the gate-induced hysteresis as a function of the gate voltage range and the sweeping time, respectively. We observe that $H_{W}$ linearly increases with the $V_{gs}$ sweeping range while it has exponential dependence on the $V_{gs}$ sweeping time (see inset of Figure 3b,c). The linear behavior of $H_{W}$ with the sweeping range indicates a trapping process that is proportional to gate voltage and loads the capacitor formed by the MoS_2_ channel and the Si substrate. The exponential dependence of $H_{W}$ on the sweeping time [63], (time constant approximately 9 min), reveals a prevalent role of slow trap states related to either MoS_2_ or SiO_2_ defects compared to the contribution of MoS_2_ defects or MoS_2_/SiO_2_ interface (fast) traps [64,65]. These results are in agreement with previous experiments on similar devices contacted by Ti/Au (titanium/gold) metal contacts [6,66].

We remark that the backward sweeps of Figure 3b, which are unaffected by the gate-voltage range, well overlap each other, indicating that the proposed contacting method enables highly reproducible measurements. The repeatability of the measurements is further demonstrated by the inset of Figure 3a, which shows two transfer curves measured before and after the two tips were detached and reconnected.

Considering the expression of the SS in terms of the trap ($C_{T}$) and channel depletion layer ($C_{DL}$) capacitances per unit area:

(1) $$SS\approx ln\left(10\right)\frac{kT}{q}\left(1+\frac{C_{T}+C_{DL}}{C_{SiO_{2}}}\right),$$

(where k is the Boltzmann constant, T is the temperature, and q is the electron charge) and assuming $C_{DL}$ is negligible with respect to $C_{T}$ (because of the low flake thickness), we estimated a trap state density of $D_{T}=\frac{C_{T}}{q^{2}}\approx 4.5\times 10^{12}{\mathrm{eV}}^{-1}{\mathrm{cm}}^{-2}$, a value consistent with existing data [6,67].

We also analyzed the effects on the transfer characteristics of electron-beam irradiation. The irradiation was performed at the electron-beam energy of 10 keV (energy typically used for SEM imaging) for an exposure time of 51 s and with a constant beam current of 0.2 nA. In Figure 3d, we report the forward and backward transfer characteristics before and after the irradiation. After the irradiation, the curve shows a left shift, which reveals an increased n-type doping of the MoS_2_. Such doping is due to positive charge accumulation from beam-induced electron-hole pair generation in SiO_2_ [66,67].

**Figure 4:**
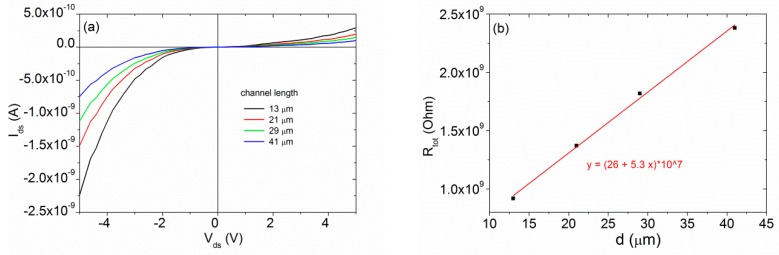
(**a**) $I_{ds}\;\;V_{ds}$ characteristics at the floating back gate as a function of the channel length. (**b**) TLM plot of $R_{tot}\;\mathrm{vs}.\;d$. The red line represents the linear fit of experimental data.

In order to study the properties of the MoS_2_/W-tip interface and estimate the contact resistance, we varied the distance between the two tips to apply the Transfer Length Method (TLM) [68]. In Figure 4a, we show the $I_{ds}-V_{ds}$ characteristics at the floating back gate as a function of the channel length. Since the characteristics have non-linear behavior, we estimated the total dynamic resistance from the linear fit of the curves in a small range of $V_{ds}$ around the value −5V. The total resistance for the two-probe configuration can be written as the sum of the channel resistance and the contact resistances, which we assumed for a rough estimation to have the same value for the two tips, i.e., $R_{tot}=\;2R_{c}+R_{ch}$. Writing $R_{ch}$ as $\frac{R_{s}}{W}d$, where d is the contacts separation, the previous becomes [69,70]:

(2) $$R_{tot}\;=\;2R_{c}+\frac{R_{s}}{W}d,$$

where $R_{s}$ is the MoS_2_ sheet resistance and W the tips diameter (≈200 nm).

From the linear fit of $R_{tot}\;\mathrm{vs}.\;d$ (see Figure 4b), we extracted the specific area contact resistivity $\rho_{c}\approx 4\;\times 10^{-2}{\;\Omega \mathrm{cm}}^{2}\;$from the intercept with *y*-axis and $R_{s}\approx 10^{8}\;\Omega /□$ from the slope. The obtained $\rho_{c}$ value is comparable to that achieved with Au contacts [71], revealing that the W-tips can form good contacts with the MoS_2_ flake. Instead, the $R_{s}$ value is about four orders of magnitude higher than the values achieved in other works [69], likely caused by a possible oxidation of the flake for a long exposure to air or by the low quality of the MoS_2_/SiO_2_ interface, which can be further optimized.

### 3.2. Field Emission Characterization

**Figure 5:**
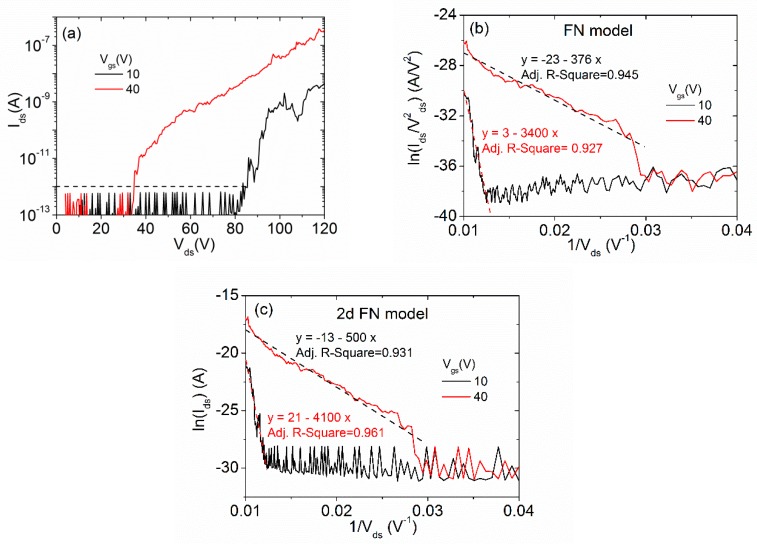
(**a**) FE current measured with the W-tip at a distance of d≈900 nm from the MoS_2_ flake and at a given back-gate voltage on a semi-logarithmic scale. (**b**) Experimental data plotted with the Fowler–Nordheim model and their linear fitting (dash lines). (**c**) Experimental data plotted with the 2D Fowler–Nordheim model [77] and their linear fitting (dash lines).

The n-type doping and the geometrical shape of the MoS_2_ flake [72] are excellent prerequisites for FE experiments. The FE measurements were implemented with a W-tip (anode) on the top of the MoS_2_ flake (cathode) in non-physical contact at a distance of 900 nm and we varied the gate voltage to observe a possible modulation of the FE current. In Figure 5a, we report FE characteristics (current−voltage), on a semi-logarithmic scale, in the voltage bias range from 5 to 120 V at given $V_{gs}$ values. Over the setup sensitivity limit of $10^{-13}\;A$, we observed an exponential increase of the current up to seven orders of magnitude as the applied voltage increased. The fluctuations and drops of the FE current can be attributed to the atomic modification of the flake edge, such as oxide or adsorbates removal by joule heating. For this reason, the initial sweep has an electrical conditioning effect and the following sweep results are smoother [39,73].

Remarkably, the FE current measured at $V_{gs}=40\;V$ is significantly higher than the one at $V_{gs}=10\;V$. An increased gate voltage enhances the n-doping of the flake and favors field emission, similarly to what has been reported in WSe_2_ vertical field emission transistors [73].

The FE current can be analyzed using the traditional Fowler–Nordheim (FN) theory [74] based on the following equation:

(3) $$I_{ds}=a\frac{E_{S}^{2}}{\Phi }S\cdot exp\left(-b\frac{\Phi^{\frac{3}{2}}}{E_{s}}\right),$$

where Φ is the work function of the emitter, S is the emitting surface, $E_{S}$ is the local electric field, and a and b are constants. The local electric field is $E_{s}=\beta \;V_{ds}/d$, where $V_{ds}$ is the applied potential, d is the cathode–anode separation distance, and β is the so-called field enhancement factor. When Φ is expressed in eV, S in ${\mathrm{cm}}^{2}$, and $E_{s}$ in V/cm, the constants a and b are $1.54\;\times \;10^{-6}{\;\mathrm{AV}}^{-2}\mathrm{eV}$ and $6.83\;\times \;10^{7}{\;\mathrm{Vcm}}^{-1}{\mathrm{eV}}^{-3/2}$, respectively. According to FN theory, $ln\left(I_{ds}/V{\;}_{ds}^{2}\right)$ vs. $1/V_{\mathrm{ds}}$ is a straight line (known as the FN plot), whose slope can be used to estimate β. Figure 5b shows the corresponding FN plots to the curves in Figure 5a and their linearity confirms that the measured currents are governed by FN tunneling. At the back-gate voltage $V_{gs}=40\;V$, we estimated a value of the turn-on field (here defined as the applied field necessary to extract a current of 10 pA) of $E_{on}=40{\;V\mu m}^{-1}$, and, assuming a MoS_2_ work function of Φ=5.15 eV [75], from the linear fitting we can estimate a maximum value of β≈200, consistent with existing data at similar cathode–anode distances [67]. The obtained $E_{on}$ value can be considered a good result compared to what has been obtained with similar measurement setups on other layered materials [12,42,67].

We note that it has been recently suggested that the FN equation should be modified to account for the 2D nature of the emitting material [73,76,77]. A new model with $I_{ds}\propto exp\left(-c\frac{\Phi^{\frac{3}{2}}}{E_{s}}\right)$, where *c* is a constant, here referred to as the 2D FN model, has been proposed [77]. The fit of the 2D FN model, shown in Figure 5c, yields a slightly better adjusted R-squared. However, the difference from the Equation (3) model is not significant and, based on the present measurements, we cannot make any sound conclusions in favor of this new model.

## 4. Conclusions

In conclusion, we proposed an easy and fast method to contact 2D flakes by the gentle touch of piezoelectric-driven tungsten tips inside a SEM chamber. We applied this method to a MoS_2_ monolayer flake that we fully characterized with measurements of parameters such as mobility, hysteresis, electron-beam effect, field emission, and so on.

Despite the facile contacting, we obtained good specific area contact resistivity $\rho_{c}\approx 4\;\times 10^{-2}\;{\Omega \mathrm{cm}}^{2}$, similar to the case of MoS_2_ devices with metal contacts deposited using EBL techniques. We also demonstrated that the direct contacting by metallic nanoprobes allows the complete electrical characterization of the transport properties of 2D FETs. In particular, we reported a complete study of the hysteresis observed in the transfer characteristics. We demonstrated that the hysteresis width has a linear dependence on the gate voltage range and an exponential dependence (with a characteristic time of 9 min) on the sweeping time. Moreover we achieved a significant gate-controlled field emission current from the edge part of the MoS_2_ flake under the application of a moderate turn-on field of approximately 40 V/μm with a field enhancement factor of approximately 200.

We have so proved that the use of tips directly on the flake, instead of metal leads, equally allows a rapid as well as a deep and complete investigation of the physical properties of the devices, avoiding an expensive and delicate EBL or optical lithography/lift-off processes.
